# Progressive Neurosarcoidosis Presenting as Idiopathic Hypopituitarism With Atypical Radiological Features for 13 Years

**DOI:** 10.7759/cureus.73575

**Published:** 2024-11-13

**Authors:** Muhammad T Younas, Jane Dale, Maryam Khan

**Affiliations:** 1 Medicine, Russells Hall Hospital, Dudley, GBR; 2 Diabetes and Endocrinology, Russells Hall Hospital, Dudley, GBR; 3 Psychology, Szkoła Wyższa Psychologii Społecznej (SWPS) University, Wrocław, POL

**Keywords:** angiotensin-converting enzyme (ace), empty sella, neurosarcoidosis, panhypopituitarism, pituitary gland

## Abstract

Sarcoidosis is an immune-mediated multisystem condition of unknown etiology, characterized by non-caseating granulomatous inflammation. While it commonly affects the lungs and the reticuloendothelial system, it can affect any organ. Most of such cases involve the central nervous system, but the condition rarely presents with symptoms related to hypothalamic-pituitary dysfunction. Neurosarcoidosis primarily targets the leptomeninges, leading to infiltration of the hypothalamus and pituitary gland by granuloma with deficiencies in luteinizing hormone (LH), follicle-stimulating hormone (FSH), and growth hormone (GH) being common. Most patients suffering from sarcoidosis develop neurological manifestations within two years of diagnosis and can present with inflammation in the pituitary gland which can mimic infiltrative pituitary lesions.

We present a 54-year-old male patient, who initially presented with infertility, hypothyroidism, and growth hormone deficiency due to presumed idiopathic hypopituitarism. He had two children following gonadotropin therapy and was maintained on pituitary hormone replacement. After 13 years, he developed further symptoms of neurosarcoidosis including cerebellar infarction, optic neuritis, and paralysis in lower limbs and later developed systemic sarcoidosis including erythema nodosum and cervical lymphadenopathy. Initially, his MRI of the brain showed a decrease in the size of the pituitary gland in 2004, and there were no other features to suggest a systemic illness. A repeat MRI of the brain in 2019 showed an empty sella. His chest X-ray was normal, T-SPOT.TB was negative, and serum angiotensin-converting enzyme (ACE) was undetectable, but eventually, a lymph node biopsy confirmed features of sarcoidosis. Unfortunately, his condition has progressed despite high-dose steroid therapy and methotrexate.

This case emphasizes the need for a thorough re-examination of features of neurosarcoidosis in cases of apparently idiopathic panhypopituitarism, to identify patients developing further complications, even after many years. Obtaining a tissue diagnosis is often difficult, and systemic features may be absent. Prospective studies are needed to establish a more uniform strategy for managing hypothalamic-pituitary neurosarcoidosis and identifying factors that predict treatment outcomes.

## Introduction

Sarcoidosis is an immune-mediated multisystemic inflammatory condition established with a propensity to infect any system of the body [1]. The scientific estimation reveals that every 3-10 per 100,000 Caucasians and 35-80 per 100,000 African Americans suffer from this condition with a small predominance of female sample [2]. Further, the lungs were reportedly the most affected organs in the cases. However, other organs such as the eyes, skin, liver, lymph nodes, and central nervous system (CNS) may also be affected. In particular, due to the sarcoid granulomas, invasion of the CNS or peripheral nervous system is referred to as neurosarcoidosis [2]. It occurs in fewer than 5-10% of the patients diagnosed with systemic sarcoidosis, whereas neurosarcoidosis without systemic involvement is one of the rarest conditions [3]. We present a case with neurosarcoidosis causing hypopituitarism initiated through pituitary gland complications. Systemic features of sarcoidosis were manifested 13 years after the sequential development of hypopituitarism.

This case report was presented as a poster at the Joint UK- Irish Endocrine Meeting in Belfast on October 14-15, 2024.

## Case presentation

**Figure 1:**
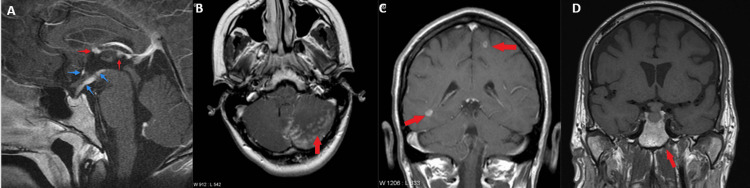
MRI showing the pituitary stalk (A), leptomeninges (B), brain parenchymal (C), and pituitary gland (D) revealing infiltrative lesions of neurosarcoidosis in the brain

**Figure 2:**
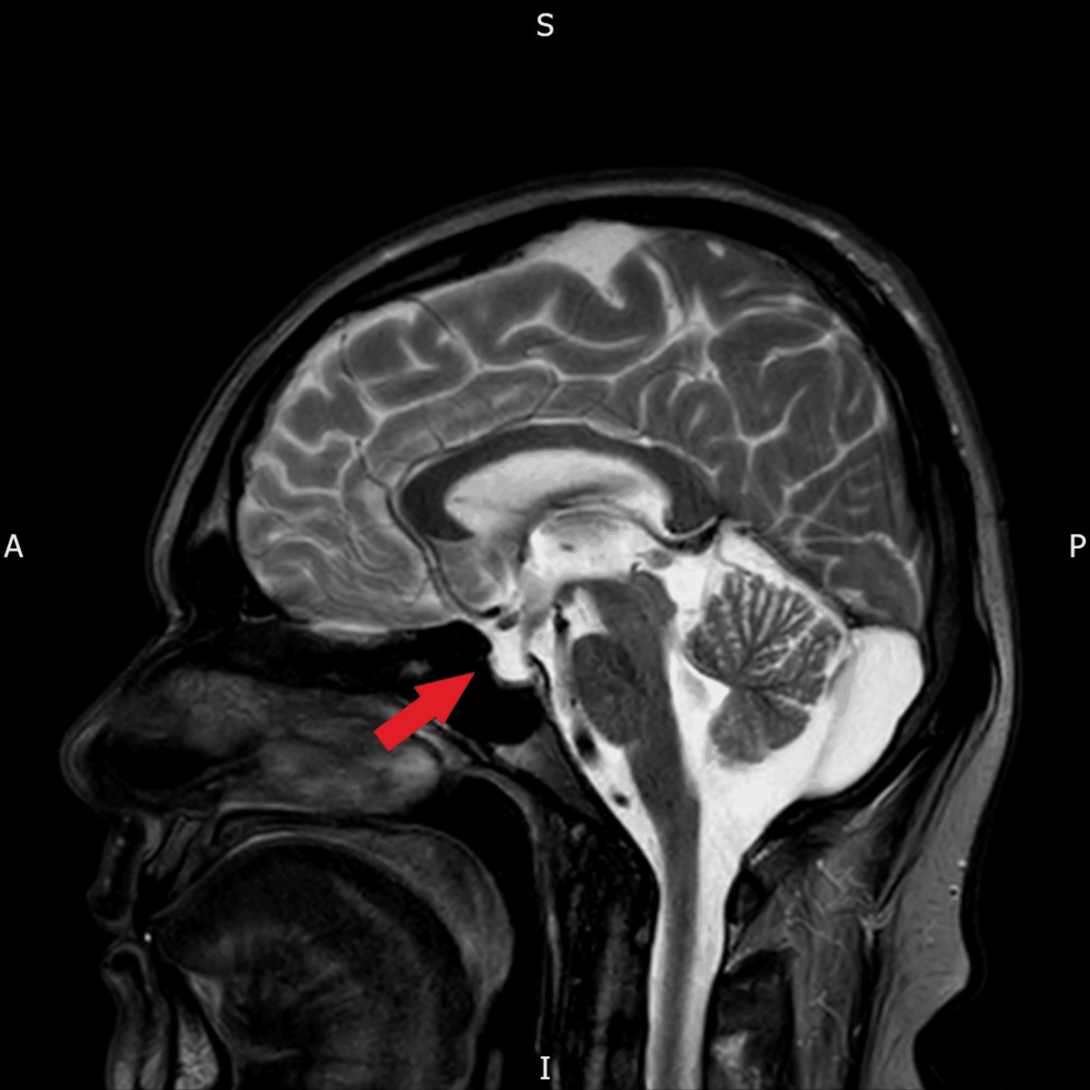
MRI of the brain showing empty sella

The MR images presented in Figure 1 reveal possible infiltrative lesions of neurosarcoidosis in the brain which can involve the leptomeninges, brain parenchyma, pituitary stalk, or pituitary gland. Many patients diagnosed with sarcoidosis develop neurosarcoidosis with neurological manifestations within two years of the diagnosis. Only a small number of cases have been reported up till now in which pituitary involvement was before the systemic manifestation of sarcoidosis. We present the case of a 54-year-old male patient diagnosed with neurosarcoidosis, initiated through hypopituitarism and afterwards leading to the development of the symptoms of sarcoidosis. At the age of 34, he was referred to the endocrine clinic with extreme tiredness, decreased libido, and infertility. Investigations showed hypogonadotropic hypogonadism with follicle-stimulating hormone (FSH) <0.1 IU/L (1.5-12.4 IU/L), luteinizing hormone (LH) <0.2 mIU/L (1.7-8.6 mIU/L), and testosterone 7.5 nmol/L (10-35 nmol/L). He was started on testosterone replacement therapy. Initially, all anterior pituitary hormonal profiles appeared normal. However, an MRI conducted in 2004 revealed a slight reduction in the size of the pituitary gland, and by 2019, the MRI indicated an empty sella, as illustrated in Figure 2. This progression underscores the importance of ongoing monitoring and attention to changes in pituitary health.

**Figure 3:**
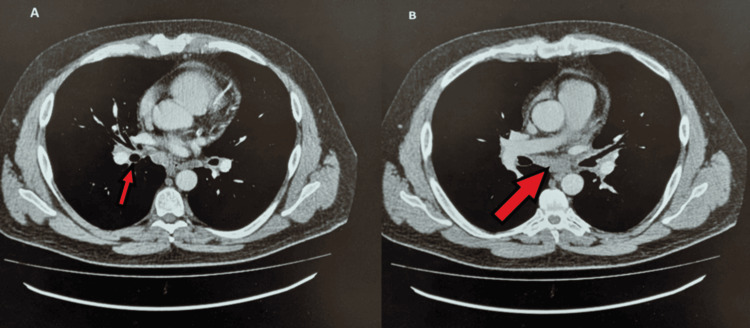
CT of the chest showing mediastinal (A) and hilar lymph nodes (B)

The patient's symptoms of tiredness and lethargy persisted. After one year, his repeat hormonal profile showed a decrease in growth hormone (GH) <0.05 mIU/L (0.5-5 mIU/L) and insulin-like growth factor-1 (IGF-1) 6.9 nmol/L (8.5-27.3 nmol/L), and its replacement was started. He was closely observed, and in the subsequent two years, he developed secondary hypothyroidism with thyroid-stimulating hormone (TSH) 0.20 mIU/L (0.27-4.20 mIU/L), free thyroxine (FT4) 10.7 pmol/L (12-22 pmol/L), and free triiodothyronine (FT3) 2.6 (3.1-6.8 pmol/L) and was started on levothyroxine. His symptoms of tiredness improved, and his short synacthen test remained normal (0 minutes 378, 30 minutes 698, and 60 minutes 672) throughout this time. Meanwhile, he had the birth of a child; everything seemed to be moving on the right path. After about eight years, he suddenly developed a stroke, and after that, he had a significant weight loss for which a workup was done and a CT of the thorax and abdomen showed multiple lymph nodes in the cervical, perihilar, and mesenteric region, as shown in Figure 3.

**Figure 4:**
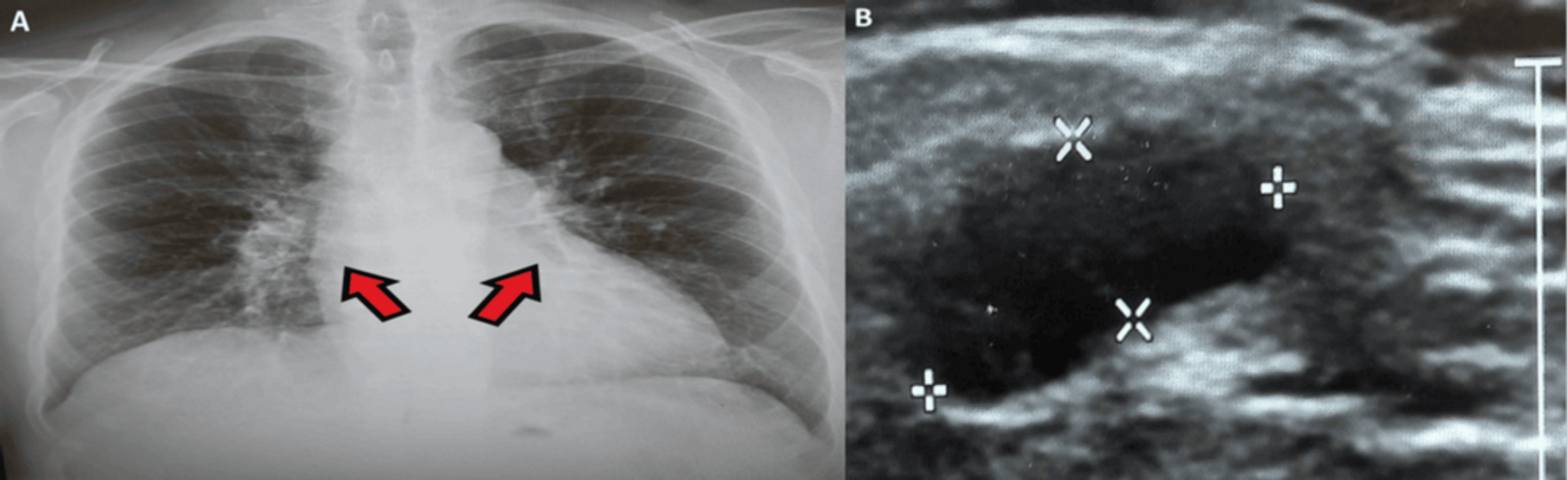
Patient's chest X-ray showing hilar prominence (A) and ultrasound of the neck showing cervical lymph nodes (B)

The patient also developed red indurated lesions on his legs which resolved on its own. A lymph node biopsy showed non-caseating granulomas raising the suspicion of tuberculosis (TB) and sarcoidosis, and he was started on anti-TB drugs by the respiratory team. His ultrasound of the neck showing cervical lymph nodes and chest X-rays are shown in Figure 4. His ACE levels were normal <12 U/L (20-70 U/L) and T-SPOT.TB was negative. Meanwhile, he developed sudden blindness in the right eye and paralysis in his legs with loss of bowel and bladder control signifying neurosarcoidosis in 2018. He was started on IV steroid pulse therapy, and afterwards, he was started on a maintenance dose of steroids. The treatment was accordingly planned and progressively modified over time. The current treatment includes hydrocortisone 10+5+5 mg, testosterone gel, clopidogrel 75 mg OD, folic acid, levothyroxine 125 micrograms OD, lansoprazole, and methotrexate 20 mg once weekly. This presents improved conditions including blood pressure 118/80, weight 106.1 kg, pulse rate 68 bpm, free T4 18.1 (12-22 pmol/L), IGF-1 13 (8.5-27.3 nmol/L), free T3 4 (3.1-6.8 pmol/L) (optimal replacement), cortisol 347 (normal >200), ACE 76 (20-70 U/L), testosterone 16.3 (8.6-29 nmol/L), LH <1 (1.7-8.6 IU/L), prolactin 377 (86-324 mu/L), prostate-specific antigen (PSA) 0.179 (0-4 ug/L), and HbA1c 53 (43-58 mmol) (improved and on target). Further, the patient was feeling well on his last visit, he had regained his weight, and hormonal replacement was optimal. The patient reported that he was feeling the best he has felt for years, and therefore, the physician had not made any changes to his treatment.

## Discussion

Neurosarcoidosis is a rare condition observed in patients with sarcoidosis accompanied by various neurological challenges like panhypopituitarism. The symptomology of neurosarcoidosis varies based on specific anatomic structures being affected by sarcoid lesions [2]. Moreover, in a relatively smaller number of cases, approximately 5-10% of sarcoidosis patients develop neurologic manifestations, whereas only 1% of sarcoidosis patients develop neurosarcoidosis [3]. Initially, the post-mortem explorations from patients having hypothalamic-pituitary failure suggested partial or entire tissue destruction resulting from the sarcoid infiltration in either the pituitary gland or hypothalamus [4]. Consequently, in recent investigations, it has been declared that hypothalamic infiltration primarily causes panhypopituitarism which provides an insightful understanding of the underlying mechanism of the condition at hand [5]. At times, it looks like a pituitary mass which is generally rare [6]. In such a situation, one of the necessities is to undergo a biopsy along with historical confirmation of the condition. Also, the occurrence of endocrine complications is reportedly estimated as 2-26% which can lead to numerous conditions like diabetes insipidus, amenorrhea, and galactorrhea. However, impaired thermoregulation is relatively less frequent in cases of panhypopituitarism [3,5]. We found the consistent features in the presented case.

At first, for the identification of sellar lesions, there are multiple possible diagnoses to look up for such as pituitary tumors, germ cell tumors, etc., whereas the diagnosis of neurosarcoidosis is challenging in terms of extensive investigation through electromyography (EMG), nerve conduction studies, cerebrospinal fluid (CSF) findings, biopsy results, and the patient's history along with physical examinations. Also, a lot of neurological complications not only look alike but also can be comorbid with neurosarcoidosis. Thus, biopsy having non-caseating granulomas would specifically work in such a situation. Having said that, it is imperative to note that a biopsy is not always practical and a safe enough option. Likewise, beyond histology, many factors like CSF results or MRI elaborate the diagnosis to a sufficient extent [7,8]. Abnormalities in the CSF and lymphocytosis in the CSF of individuals with neurosarcoidosis are also evident from the medical examinations in the due course. Almost 55% estimation is available for the cases of lymphocytosis in CSF [8]. Further, MRI is very sensitive but not specific in the detection of abnormalities in this condition. Moreover, the observation of periventricular T2-weighted hyperintense lesions resembling multiple sclerosis and impacting the corpus callosum is very common [9]. Therefore, it's essentially obvious that half of the patients with panhypopituitarism sarcoidosis appear normal in radiological examination [10].

This all demands a timely diagnosis, and the provision of corticosteroids and immunosuppressants would not only serve as a life-saving step but also control the inflammatory aspects of the condition. Further, long-term, follow-up with recurrent imaging and immunologic agents when the problem persists like TNF-alpha inhibitor would essentially be a promising move. All in all, this presentation serves two key purposes. Firstly, it demonstrates the fact that timely investigation of neurosarcoidosis for cases with panhypopituitarism will address the issue proactively and prevent the patient from developing further complications. It is the hallmark of this attempt to emphasize this point because once neurosarcoidosis affects the endocrine system, then these treatments might not be helpful in the restoration of pituitary hormones to their normal efficiency. Lastly, with this evidence-based case report, we tried to add up the scientific knowledge of the condition to help physicians be vigilant during such procedures.

## Conclusions

We present one of the rarest conditions of panhypopituitarism essentially uncovering the underlying cause, neurosarcoidosis with an evidence-based claim that its prompt examination and exploration would not only benefit the patient by controlling the deteriorating pituitary conditions but also limit the inflammatory aspects of the disease. Therefore, our attempt is an insightful guide for physicians working on the diagnosis and management of panhypopituitarism and neurosarcoidosis.

## References

[1] Martin-Grace J, Murialdo G, Tamagno G (2015). Hypothalamic-pituitary alterations in patients with neurosarcoidosis. EMJ Neurol.

[2] Ibitoye RT, Wilkins A, Scolding NJ (2017). Neurosarcoidosis: a clinical approach to diagnosis and management. J Neurol.

[3] Hebel R, Dubaniewicz-Wybieralska M, Dubaniewicz A (2015). Overview of neurosarcoidosis: recent advances. J Neurol.

[4] BL VR, RO SL (1952). Sarcoid-like granulomata of the pituitary gland; a cause of pituitary insufficiency. AMA Arch Intern Med.

[5] Stuart CA, Neelon FA, Lebovitz HE (1978). Hypothalamic insufficiency: the cause of hypopituitarism in sarcoidosis. Ann Intern Med.

[6] Guoth MS, Kim J, de Lotbiniere AC, Brines ML (1998). Neurosarcoidosis presenting as hypopituitarism and a cystic pituitary mass. Am J Med Sci.

[7] Valeyre D, Prasse A, Nunes H, Uzunhan Y, Brillet PY, Müller-Quernheim J (2014). Sarcoidosis. Lancet.

[8] Zajicek JP, Scolding NJ, Foster O (1999). Central nervous system sarcoidosis--diagnosis and management. QJM.

[9] Lacomis D (2011). Neurosarcoidosis. Curr Neuropharmacol.

[10] Hoyle JC, Jablonski C, Newton HB (2014). Neurosarcoidosis: clinical review of a disorder with challenging inpatient presentations and diagnostic considerations. Neurohospitalist.

